# Supplementary material for: Cerebrospinal fluid endo-lysosomal proteins as potential biomarkers for Huntington’s disease
Source: PLoS One. 2020 Aug 17;15(8):e0233820. doi: 10.1371/journal.pone.0233820 (PMC7430717; doi:10.1371/journal.pone.0233820)
Supplement: S1 Table — Information pertaining to all CSF endo-lysosomal proteins used in the study. (PDF) [file pone.0233820.s004.pdf]

**Protein Name**	**Abbreviation**	**UniProtKB Accession**	**Peptide**	**Sequence**	**Internal Standard Mixture Concentration, M**	**Tryptic Peptide Isolation, *m/z***	**Isotope-Labelled Peptide Isolation, *m/z***	**Charge, *z***	**Polarity**	**Normalized Collision Energy**	**Retention Time, min**	**Fragment Ions**	**Peptide Ratio Repeatability, CV (%)**	**Peptide Ratio Intermediate Precision, CV (%)**	**Mean Peptide Ratio Repeatability, CV (%)**	**Mean Peptide Ratio Intermediate Precision, CV (%)**
**AP-2 complex subunit beta**	AP2B1	P63010	AP2B1_712-719	AVWLPAVK	2.86E-10	442.77346	446.78056	2	Positive	22	24.5	y4-y6	14.1	16.9	13.0	16.5
			AP2B1_835-842	QVFLATWK	2.86E-10	497.28166	501.28876	2	Positive	20	24.9	y3, y4, y6	14.0	20.1		
			AP2B1_868-878	LQNNNVYTIAK	1.91E-10	639.84596	643.85306	2	Positive	24	12.25	y4, y9	12.9	14.4		
**Amyloid beta A4 protein**	APP	P05067	APP_289-301	EVCSEQAETGPCR	1.73E-09	762.31656	767.32066	2	Positive	24	5.4	y3-y11	1.8	2.7	2.1	2.1
			APP_439-450	VESLEQEAANER	1.95E-09	688.32826	693.33246	2	Positive	24	11.1	y3-y5, y7-y10	4.7	5.2		
**Bovine serum albumin**	BSA	P02769	BSA_421-433	LGEYGFQNALIVR	1.91E-07	740.90126	745.90536	2	Positive	24	35.9	y3-y12	6.0	10.4	6.0	10.4
**Complement component C9**	C9	P02748	C9_146-154	VVEESELAR	2.88E-09	516.77186	521.77606	2	Positive	18	7.5	y3, y5-y7	2.0	3.5	2.0	2.4
			C9_186-194	DGNTLTYYR	4.50E-09	552.26166	557.26586	2	Positive	22	12.2	y3-y6	2.7	2.7		
**Cathepsin B**	CTSB	P07858	CTSB_210-220	ICEPGYSPTYK	8.34E-10	658.30526	662.31236	2	Positive	26	13.5	y4, y5, y7-y9	12.1	12.1	9.1	10.1
			CTSB_58-71	LCGTFLGGPKPPQR	3.10E-09	510.27474	513.61084	3	Positive	30	17.5	y3, y4, y6-y9	12.9	18.3		
			CTSB_80-87	LPASFDAR	8.34E-10	439.23216	444.23636	2	Positive	24	10.9	y5-y7	6.4	6.4		
**Cathepsin D**	CTSD	P07339	CTSD_112-122	LLDIACWIHHK	3.69E-09	469.58504	472.25644	3	Positive	26	31.15	y3, y5-y7	13.0	14.8	9.7	11.7
			CTSD_349-357	LSPEDYTLK	2.82E-09	533.77666	537.78376	2	Positive	28	18.1	y3-y7	4.8	5.3		
			CTSD_55-72	YSQAVPAVTEGPIPEVLK	1.19E-09	950.01696	954.02406	2	Positive	24	39.05	y5, y8, y13	13.2	16.4		
**Cathepsin F**	CTSF	Q9UBX1	CTSF_103-116	TLLCSFQVLDELGR	9.53E-10	826.42946	831.43366	2	Positive	26	48.6	y4-y11	11.2	15.0	10.1	12.2

			CTSF_236-245	FSDLTEEEFR	2.15E-10	637.29066	642.29476	2	Positive	20	21.7	y4-y9	4.8	6.4		
			CTSF_266-278	SVGDLAPPEWDWR	1.69E-10	764.86486	769.86896	2	Positive	24	39.2	y7, y8	14.5	18.9		
			CTSF_442-450	SDVPFWAIK	3.58E-10	532.28446	536.29156	2	Positive	24	32.4	y4-y6	14.0	14.0		
**Cathepsin L1**	CTSL	P07711	CTSL_105-116	VFQEPLFYEAPR	3.58E-10	748.88246	753.88666	2	Positive	28	33.9	y5-y10	10.2	12.3	10.2	12.3
**Cathepsin Z**	CTSZ	Q9UBR2	CTSZ_261-270	NSWGEPWGER	5.96E-10	609.77016	614.77436	2	Positive	22	19.55	y3, y5, y7, y8	7.9	10.2	3.2	6.2
			CTSZ_39-47	GDGLAPLGR	5.96E-10	428.73766	433.74176	2	Positive	22	11.05	y4, y5	6.4	7.2		
**Dipeptidyl peptidase 2**	DPP2	Q9UHL4	DPP2_113-123	SLPFGAQSTQR	2.38E-10	596.80936	601.81346	2	Positive	28	13.6	y7-y9	11.8	15.0	8.0	9.9
			DPP2_40-47	LDHFNFER	1.67E-10	360.17514	363.51124	3	Positive	24	12.75	y3-y5	9.4	9.8		
			DPP2_449-462	ASHPEDPASVVEAR	3.47E-10	489.24064	492.57674	3	Positive	24	10.8	y3, y4, y6	4.7	6.7		
**Tissue alpha-L-fucosidase**	FUCA1	P04066	FUCA1_114-130	FFHPEEWADLFQAAGAK	3.58E-09	655.64934	658.32074	3	Positive	20	47.4	y5-y9	14.0	18.5	12.2	16.6
			FUCA1_163-173	DLVGELGTALR	1.43E-10	572.82186	577.82606	2	Positive	18	37.5	y5, y6, y8, y9	11.3	13.7		
			FUCA1_344-354	DGLIVPIFQER	1.67E-10	644.35866	649.36276	2	Positive	28	42.3	y4, y6-y8	12.6	16.6		
**Ganglioside GM2 activator**	GM2A	P17900	GM2A_170-179	IESVLSSSGK	2.40E-09	504.27426	508.28136	2	Positive	26	7.65	y4-y9	5.5	9.6	4.8	4.8
			GM2A_89-96	EVAGLWIK	4.40E-09	458.76846	462.77556	2	Positive	18	23.65	y3-y6	6.4	8.7		
**Beta-hexosaminidase subunit beta**	HEXB	P07686	HEXB_285-300	VLPEFDTPGHTLSWGK	2.38E-10	595.63854	598.30994	3	Positive	28	33.25	y4, y6, y9	14.7	19.1	13.9	18.3
			HEXB_391-400	VLDIIATINK	9.53E-10	550.83956	554.84666	2	Positive	20	31.25	y6-y8	13.4	17.7		
**Lysosome-associated membrane glycoprotein 1**	LAMP1	P11279	LAMP1_138-146	TVESITDIR	7.25E-10	517.77966	522.78386	2	Positive	20	14.75	y3-y5, y7	2.6	4.7	7.5	8.9
			LAMP1_327-337	ALQATVGNSYK	2.38E-10	576.80626	580.81336	2	Positive	24	8.45	y5-y8	12.2	12.7		
			LAMP1_357-363	VWVQAFK	1.91E-10	439.75006	443.75716	2	Positive	26	18	y3-y6	9.4	10.9		

**Lysosome-associated membrane glycoprotein 2**	LAMP2	P13473	LAMP2_133-144	GILTVDELLAIR	3.81E-09	657.39526	662.39936	2	Positive	26	49.45	y3-y10	15.8	15.8	10.4	11.9
			LAMP2_145-152	IPLNDLFR	4.65E-09	494.78456	499.78876	2	Positive	28	31.35	y3-y7	12.9	16.7		
			LAMP2_153-161	CNSLSTLEK	2.28E-08	526.75796	530.76506	2	Positive	22	7.65	y4-y8	4.8	7.2		
			LAMP2_281-289	YLDFVFAVK	6.07E-09	551.80246	555.80956	2	Positive	22	40.5	y4, y6-y8	13.1	21.0		
**Lysozyme C**	LYZ	P61626	LYZ_52-59	WESGYNTR	2.60E-09	507.22766	512.23176	2	Positive	22	5.8	y3, y5-y7	2.8	7.2	5.0	10.1
			LYZ_69-80	STDYGIFQINSR	1.12E-08	701.34376	706.34786	2	Positive	22	28.2	y4-y8	8.9	14.0		
**Transcobalamin-2**	TCN2	P20062	TCN2_300-313	TYIDLIFPDCLAPR	7.15E-10	847.93436	852.93856	2	Positive	28	51.6	y5, y8-y11	13.9	17.1	13.7	14.3
			TCN2_393-399	EFWQLLR	2.92E-10	496.77146	501.77566	2	Positive	20	35.65	y3-y5	13.5	13.7		
			TCN2_45-59	LSLEHLNPSIYVGLR	4.84E-10	571.32264	574.65874	3	Positive	26	38.8	y4-y6	14.7	14.7		
**Tripeptidyl-peptidase 1**	TPP1	O14773	TPP1_246-259	LFGGNFAHQASVAR	3.58E-10	492.58974	495.92584	3	Positive	28	17.6	y5, y7, y8	14.2	18.7	13.1	17.8
			TPP1_507-520	LYQQHGAGLFDVTR	4.54E-10	535.94404	539.28014	3	Positive	24	21.1	y3-y5, y7-y10	13.0	17.6		
			TPP1_61-78	LSELVQAVSDPSSPQYGK	3.58E-10	953.48346	957.49056	2	Positive	24	27.4	y5, y8, y10-y12	12.7	17.2		
**Ubiquitin**	Ubiquitin	P0CG48	Ubiquitin_12-27	TITLEVEPSDTIENVK	2.00E-08	894.96716	933.59626	2	Positive	25	32.6	y9-y11	10.8	14.6	11.4	15.1
			Ubiquitin_64-72	ESTLHLVLR	2.00E-08	356.87834	372.59754	3	Positive	25	16.6	y3-y5	12.5	16.1		
