# Supplementary material for: Cerebrospinal fluid endo-lysosomal proteins as potential biomarkers for Huntington’s disease
Source: PLoS One. 2020 Aug 17;15(8):e0233820. doi: 10.1371/journal.pone.0233820 (PMC7430717; doi:10.1371/journal.pone.0233820)
Supplement: S2 Table — Differences in scores across disease stage. P-values were Bonferroni-corrected and generated from general linear models. CAG was included in the model when assessing differences between manifest and premanifest HD mutation carriers. (PDF) [file pone.0233820.s005.pdf]

**Principal Components**	**Adjusted for**	**ANOVA *p* value**	**Controls vs Premanifest *p* value**	**Manifest vs Premanifest *p* value**
PC1	Age	0.53	0.66	0.60
	Age and CAG	NA	NA	0.37
PC2	Age	0.86	1.00	1.00
	Age and CAG	NA	NA	0.59
PC3	Age	0.47	0.56	1.00
	Age and CAG	NA	NA	0.69
