# Supplementary material for: Cerebrospinal fluid endo-lysosomal proteins as potential biomarkers for Huntington’s disease
Source: PLoS One. 2020 Aug 17;15(8):e0233820. doi: 10.1371/journal.pone.0233820 (PMC7430717; doi:10.1371/journal.pone.0233820)
Supplement: S3 Table — The relationships between principal components 1 (PC1), 2 (PC2) and 3 (PC3) and Disease Burden Score (DBS) were computed using Pearson’s correlation with unadjusted values shown. Relationships with composite Unified Huntington’s Disease Rating Scale (cUHDRS), Total Functional Capacity (TFC), Total Motor Score (TMS), Symbol Digit Modalities Test (SDMT), and Stroop Word Reading (SWR) were assessed using Pearson’s partial correlation controlling for age, and age and CAG. Correlation coefficients and 95% confidence intervals were computed using bootstrap testing with 1000 repetitions. Results shown are unadjusted for multiplicity. Bold text indicates significance at p<0.05. (PDF) [file pone.0233820.s006.pdf]

Principal Components	DBS *r* (95% CI)	Adjusted for	cUHDRS *r* (95% CI)	TFC *r* (95% CI)	TMS *r* (95% CI)	SDMT *r* (95% CI)	SWR *r* (95% CI)
PC1	0.03 (-0.25, 0.27)	Age	0.21 (-0.09, 0.47)	0.17 (-0.07, 0.40)	-0.20 (-0.43, 0.06)	0.22 (-0.09, 0.49)	0.19 (-0.11, 0.44)
		Age and CAG	0.23 (-0.06, 0.50)	0.17 (-0.07, 0.41)	-0.21 (-0.44, 0.04)	0.24 (-0.06, 0.54)	0.20 (-0.12, 0.47)
PC2	0.03 (-0.24, 0.23)	Age	0.20 (-0.08, 0.44)	0.21 (-0.02, 0.41)	-0.17 (-0.39. 0.06)	0.23 (-0.09, 0.50)	0.16 (-0.14, 0.39)
		Age and CAG	0.25 (-0.03, 0.49)	**0.23 (0.01, 0.44)**	-0.20 (-0.42, 0.07)	0.27 (-0.02, 0.55)	0.19 (-0.11, 0.46)
PC3	0.20 (-0.02, 0.44)	Age	-0.19 (-0.42, 0.10)	**-0.28 (-0.54, 0.00)**	0.14 (-0.12, 0.34)	-0.13 (-0.37, 0.19)	-0.18 (-0.39, 0.08)
		Age and CAG	-0.10 (-0.35, 0.23)	-0.22 (-0.50, 0.09)	0.04 (-0.24, 0.28)	-0.02 (-0.27, 0.34)	-0.09 (-0.32, 0.19)
