# Supplementary material for: Cerebrospinal fluid endo-lysosomal proteins as potential biomarkers for Huntington’s disease
Source: PLoS One. 2020 Aug 17;15(8):e0233820. doi: 10.1371/journal.pone.0233820 (PMC7430717; doi:10.1371/journal.pone.0233820)
Supplement: S4 Table — Values are Pearson’s r and t-test statistic. Bold indicates significance at the p<0.05 level. (PDF) [file pone.0233820.s007.pdf]

	Age		Gender		Haemoglobin	
**Lysosomal Proteins**	***r***	***p* value**	***t***	***p* value**	***r***	***p* value**
AP2	0.20	0.39	-0.07	0.94	-0.09	0.72
APP	0.17	0.49	-0.25	0.80	0.05	0.85
C9	0.50	**0.03**	0.03	0.97	0.27	0.26
Cathepsin B	0.17	0.45	-0.99	0.33	0.004	0.98
Cathepsin L	0.15	0.52	-0.55	0.59	0.04	0.86
Cathepsin Z	0.24	0.31	-0.48	0.63	0.17	0.47
DPP2	0.13	0.58	-1.48	0.16	0.12	0.63
HEXB	-0.07	0.77	-0.38	0.70	0.03	0.91
LYZ	0.70	**<0.001**	-2.46	**0.02**	0.31	0.17
FUCA	0.23	0.35	-0.65	0.52	0.09	0.69
TCN2	0.28	0.23	-0.90	0.37	-0.16	0.49
TPP1	-0.19	0.41	0.11	0.91	-0.03	0.88
UBQ	0.34	0.14	-0.22	0.83	0.03	0.92
