# Supplementary material for: Cerebrospinal fluid endo-lysosomal proteins as potential biomarkers for Huntington’s disease
Source: PLoS One. 2020 Aug 17;15(8):e0233820. doi: 10.1371/journal.pone.0233820 (PMC7430717; doi:10.1371/journal.pone.0233820)
Supplement: S5 Table — Differences in analyte concentration across disease stage were Bonferroni-corrected and generated from general linear models controlling for age, or age and CAG. Gender was also included in the model for LYZ. Bold indicates significance at the p<0.05 level. (PDF) [file pone.0233820.s008.pdf]

Lysosomal Proteins	Adjusted for	ANOVA *p* value	Controls vs Premanifest *p* value	Manifest vs Premanifest *p* value
AP2	Age	0.91	1.00	1.00
	Age and CAG	NA	NA	0.93
APP	Age	0.23	0.90	0.20
	Age and CAG	NA	NA	0.25
C9	Age	0.64	1.00	1.00
	Age and CAG	NA	NA	0.69
Cathepsin B	Age	0.26	0.59	0.20
	Age and CAG	NA	NA	0.07
Cathepsin L	Age	0.84	1.00	1.00
	Age and CAG	NA	NA	0.53
Cathepsin Z	Age	0.10	0.08	0.13
	Age and CAG	NA	NA	**0.04**
DPP2	Age	0.89	1.00	1.00
	Age and CAG	NA	NA	0.58
HEXB	Age	0.62	0.94	0.68
	Age and CAG	NA	NA	0.21
LYZ	Age and Gender	0.48	0.83	1.00
	Age, CAG, Gender	NA	NA	0.89
FUCA	Age	0.15	0.11	0.35
	Age and CAG	NA	NA	0.14
TCN2	Age	0.47	1.00	0.47
	Age and CAG	NA	NA	0.11
TPP1	Age	0.56	0.83	0.57
	Age and CAG	NA	NA	0.17
UBQ	Age	0.57	1.00	0.63
	Age and CAG	NA	NA	0.50
