# Supplementary material for: Cerebrospinal fluid endo-lysosomal proteins as potential biomarkers for Huntington’s disease
Source: PLoS One. 2020 Aug 17;15(8):e0233820. doi: 10.1371/journal.pone.0233820 (PMC7430717; doi:10.1371/journal.pone.0233820)
Supplement: S6 Table — Associations between analyte concentration and Disease Burden Score (DBS) were assessed using Pearson’s correlation with unadjusted values displayed. Associations with composite Unified Huntington’s Disease Rating Scale (cUHDRS), Total Functional Capacity (TFC), Total Motor Score (TMS), Symbol Digit Modalities Test (SDMT), and Stroop Word Reading (SWR) were assessed using partial correlation with age, and age and CAG included in the model. For LYZ, the effects of gender were also controlled for. Significant associations are highlighted in bold. Correlation coefficients and confidence intervals were both generated using bootstrapping with 1000 repetitions. Bold indicates significance at the p<0.05 level. (PDF) [file pone.0233820.s009.pdf]

Lysosomal Proteins	DBS *r* (95% CI)	Adjusted for	cUHDRS *r* (95% CI)	TFC *r* (95% CI)	TMS *r* (95% CI)	SDMT *r* (95% CI)	SWR *r* (95% CI)
AP2	0.18 (-0.14, 0.40)	Age	0.16 (-0.10, 0.42)	0.20 (-0.04, 0.40)	-0.14 (-0.37, 0.11)	0.18 (-0.13, 0.45)	0.11 (-0.17, 0.36)
		Age and CAG	0.17 (-0.12, 0.43)	0.20 (-0.04, 0.41)	-0.13 (-0.36, 0.15)	0.18 (-0.12, 0.45)	0.10 (-0.21, 0.38)
C9	**0.36 (0.15, 0.57)**	Age	-0.23 (-0.47, 0.01)	**-0.33 (-0.54, -0.11)**	0.18 (-0.06, 0.37)	-0.17 (-0.42, 0.12)	-0.20 (-0.43, 0.04)
		Age and CAG	-0.11 (-0.40, 0.19)	-0.24 (-0.49, 0.01)	0.04 (-0.24, 0.29)	-0.03 (-0.32, 0.27)	-0.07 (-0.34, 0.23)
Cathepsin L	0.18 (-0.15, 0.39)	Age	0.15 (-0.14, 0.42)	0.11 (-0.14, 0.36)	-0.13 (-0.36, 0.10)	0.17 (-0.14, 0.46)	0.10 (-0.17, 0.36)
		Age and CAG	0.18 (-0.10, 0.45)	0.13 (-0.12, 0.37)	-0.15 (-0.38, 0.10)	0.20 (-0.09, 0.50)	0.13 (-0.18, 0.40)
Cathepsin Z	0.13 (-0.21, 0.41)	Age	0.24 (-0.08, 0.50)	0.14 (-0.13, 0.37)	-0.23 (-0.46, 0.05)	0.28 (-0.03, 0.52)	0.24 (-0.07, 0.51)
		Age and CAG	**0.28 (0.04, 0.54)**	0.14 (-0.12, 0.36)	**-0.25 (-0.47, -0.01)**	**0.31 (0.05, 0.57)**	0.28 (-0.05, 0.52)
DPP2	0.12 (-0.18, 0.35)	Age	0.05 (-0.28, 0.34)	0.04 (-0.26, 0.30)	-0.05 (-0.32, 0.30)	0.09 (-0.24, 0.40)	0.03 (-0.22, 0.32)
		Age and CAG	0.03 (-0.24, 0.34)	0.02 (-0.25, 0.29)	-0.02 (-0.28, 0.31)	0.08 (-0.22, 0.35)	0.004 (-0.26, 0.27)
LYZ	**0.40 (0.14, 0.61)**	Age and Gender	-0.08 (-0.35, 0.19)	-0.11 (-0.43, 0.24)	0.11 (-0.15, 0.37)	-0.04 (-0.30, 0.25)	-0.11 (-0.34, 0.15)
		Age, CAG, Gender	-0.03 (-0.30, 0.27)	-0.07 (-0.39, 0.17)	0.07 (-0.21, 0.34)	0.01 (-0.25, 0.33)	-0.07 (-0.32, 0.18)
TCN2	0.22 (-0.05, 0.48)	Age	0.07 (-0.19, 0.32)	0.04 (-0.25, 0.28)	-0.12 (-0.34, 0.11)	0.11 (-0.18, 0.38)	0.05 (-0.17, 0.29)
		Age and CAG	0.18 (-0.12, 0.47)	0.10 (-0.17, 0.36)	-0.22 (-0.45, 0.05)	0.21 (-0.11, 0.51)	0.14 (-0.10, 0.41)
TPP1	0.03 (-0.22, 0.24)	Age	0.20 (-0.09, 0.44)	0.12 (-0.14, 0.35)	-0.23 (-0.44, 0.01)	0.17 (-0.11, 0.43)	0.20 (-0.10, 0.47)
		Age and CAG	0.23 (-0.08, 0.48)	0.13 (-0.13, 0.36)	**-0.26 (-0.46, -0.04)**	0.19 (-0.11, 0.50)	0.23 (-0.09, 0.48)
