# Supplementary material for: Cerebrospinal fluid endo-lysosomal proteins as potential biomarkers for Huntington’s disease
Source: PLoS One. 2020 Aug 17;15(8):e0233820. doi: 10.1371/journal.pone.0233820 (PMC7430717; doi:10.1371/journal.pone.0233820)
Supplement: Attachment — Submitted filename: Response to Reviewers.docx [file pone.0233820.s011.docx]

**CEREBROSPINAL FLUID ENDO-LYSOSOMAL PROTEINS AS POTENTIAL BIOMARKERS FOR HUNTINGTON’S DISEASE.**

Lowe et al., Manuscript ID: PONE-D-20-14019

**RESPONSE TO REVIEWERS**

Dear Professor Ginsberg,

We are extremely grateful for the reviewers’ insightful and constructive comments. We have now revised our manuscript, and we hope you and they will agree it has improved it significantly. Our responses and revisions are set out below.

We hope our manuscript is now considered suitable for publication in PLOS ONE, and look forward to hearing from you.

Yours sincerely,

Mr Alexander J. Lowe

Dr Edward J. Wild FRCP

**Reviewer 1**

**Critique:**

**I have only one concern with the writing of the manuscript in the introduction, starting with line 56 suggesting there are only 2 autophagic mechanisms, macroautophagy and chaperone-mediated autophagy (CMA). Autophagy has been a very fast-moving field over the last 15 years, and the introduction cites some outdated information. Macroautophagy involves the engulfment of cargo into a double-membrane vesicle termed the autophagosome which fuses with vesicles from the endo-lysosomal compartment before becoming an autolysosome, in which its cargo is degraded. Other autophagy processes, which do not involve formation of double membrane autophagosomes, are microautophagy, endosomal microautophagy and CMA. These processes proceed with a direct engulfment of cargo into the endo-lysosomal compartment, either through production of intraluminal vesicles or direct import. The Lamp2 protein, including the Lamp-2A splice variant previously only defined in CMA, has now been found to be involved in syntaxin-17-mediated vesicle fusion in macroautophagy, while syntaxin-17 has also been shown to be required for mitochondrial single-membrane vesicle fusion to the endo-lysosomal system in microautophagy. KFERQ-tagged proteins, once thought to be only cleared by CMA have been found to be also degraded by endosomal microautophagy requiring Hsc70 for membrane-fusion events. Recent blurring of the defining lines and machinery between macroautophagy, microautophagy and CMA make understanding the autophagic alterations in PD, AD, polyQ disorders and HD more challenging, especially in light of the fact that the HTT protein itself has been found to be an autophagic scaffold, mutation of which may impair some forms of selective autophagy**

We apologise for the lack of clarity and precision in our original submission and thank the reviewer for this important steer and for taking the time to make such constructive and detailed suggestions for improving the introduction. We have updated and expanded the paragraph in question. Please see below for the changes made, starting at line 56 and ending at line 87 of the resubmitted manuscript:

“….*a lysosomal pathway that serves to eliminate toxic substances via three mechanisms, namely microautophagy, macroautophagy and chaperone-mediated autophagy (CMA) [4]. Macroautophagy involves the engulfment of cargo into a double-membrane sequestering vesicle known as an autophagosome. Following fusion with vesicles from the endo-lysosomal compartment, an autolysosome is formed in which the cargo is degraded by lysosomal hydrolases and the resultant macromolecules are released back into the cytosol [5]. Microautophagy and CMA do not involve the formation of an autophagosome, instead using direct import or intraluminal vesicle formation to engulf cargo into the endo-lysosomal compartment [6]. Despite their differences, mechanistic crossovers between the three autophagy pathways have been described. Lysosomal-associated membrane protein-2 splice variant A (LAMP-2A), previously only described in CMA, has been shown to be important for syntaxin-17 mediated vesicle fusion in macroautophagy [7]. Furthermore, syntaxin-17 is pivotal for targeting mitochondrial-derived vesicles to the endo-lysosomal compartment for degradation in microautophagy [8].*

*Macroautophagy plays a pivotal role in the clearance of aggregated proteins via aggrephagy [4,9], whereby aggregates are selectively bound to the autophagosome membrane through the action of adaptor proteins, including p62 and Alfy [10]. Autophagy disruptions have been reported in several neurodegenerative diseases including HD, in which basal autophagy appears to function normally; however, the autophagosomes are devoid of cargo, as recruitment of mHTT to the organelle fails [11–17]. Interestingly, HTT shows structural similarities to three selective autophagy proteins in yeast [18,19] and promotes selective macroautophagy in mammalian cells by mediating the binding of p62 and the autophagy-initiating kinase, UKL1 [20]. As such, it is possible that the polyglutamine expansion in HD may disrupt HTT’s role in selective autophagy [21]; however, studies have shown that autophagic clearance of aggregates can still occur despite overexpression of mHTT in mice and cellular models [22,23]. In light of HTT’s role as an autophagic scaffold protein, the mechanistic crossovers between the three pathways, and their possible contribution to neurodegeneration, we sought to study the alterations and autophagic dysfunction in HD mutation carriers and controls.*
